# High Normal Urinary Albumin–Creatinine Ratio Is Associated With Hypertension, Type 2 Diabetes Mellitus, HTN With T2DM, Dyslipidemia, and Cardiovascular Diseases in the Chinese Population: A Report From the REACTION Study

**DOI:** 10.3389/fendo.2022.864562

**Published:** 2022-05-20

**Authors:** Jie Wang, Yun Wang, Yijun Li, Ying Hu, Lingzi Jin, Weiqing Wang, Zhengnan Gao, Xulei Tang, Li Yan, Qin Wan, Zuojie Luo, Guijun Qin, Lulu Chen, Weijun Gu, Zhaohui Lyv, Yiming Mu

**Affiliations:** ^1^ Department of Endocrinology, The First Medical Center of PLA General Hospital, Beijing, China; ^2^ Department of Endocrinology, Beijing Chao-Yang Hospital, Capital Medical University, Beijing, China; ^3^ Graduate School, Chinese General Hospital, Beijing, China; ^4^ Department of International Medical Services, Peking Union Medical College Hospital, Beijing, China; ^5^ Department of Endocrinology, Shanghai National Research Centre for Endocrine and Metabolic Diseases, Ruijin Hospital, Shanghai Jiaotong University School of Medicine, Shanghai, China; ^6^ Department of Endocrinology, Dalian Central Hospital, Dalian, China; ^7^ First Hospital of Lanzhou University, Lanzhou, China; ^8^ Department of Endocrinology, Sun Yat-Sen Memorial Hospital, Zhongshan University, Guangzhou, China; ^9^ Department of Endocrinology, Southwest Medical University Affiliated Hospital, Luzhou, China; ^10^ Department of Endocrinology, First Affiliated Hospital of Guangxi Medical University, Nanning, China; ^11^ First Affiliated Hospital of Zhengzhou University, Zhengzhou, China; ^12^ Wuhan Union Hospital, Huazhong University of Science and Technology, Wuhan, China

**Keywords:** urinary albumin–creatinine ratio, hypertension, diabetes, dyslipidemia, cardiovascular diseases

## Abstract

**Background:**

Albuminuria has been widely considered a risk factor for cardiovascular diseases (CVDs), which is associated with hypertension (HTN), type 2 diabetes mellitus (T2DM), HTN with T2DM, and dyslipidemia. However, the associations between albuminuria and HTN, T2DM, HTN with T2DM, dyslipidemia, and CVDs are still unclear. Thus, this study aimed to explore the association of albuminuria thoroughly, especially within the normal range, with the abovementioned diseases in the Chinese population.

**Methods:**

This study included 40,188 participants aged over 40 years from seven centers across China. Urinary albumin–creatinine ratio (UACR) was firstly divided into the ≥30-mg/g group, indicating kidney damage, and <30-mg/g group. Furthermore, UACR was divided into five groups: the <20%, 20%–39%, 40%–59%, 60%–79%, and ≥80% groups, according to the quintile division of participants within the normal range. Propensity score matching was used to reduce bias, and multiple logistic regression models were conducted to examine the association between UACR and HTN, T2DM, HTN with T2DM, dyslipidemia, and CVDs.

**Results:**

Multivariable regression analysis revealed that UACR, even within the normal range, is significantly associated with HTN, T2DM, HTN with T2DM, dyslipidemia, and CVDs, and the association between UACR and HTN with T2DM was the most significant in model 3 even after adjusting for confounding factors (HTN: OR = 1.56 (95% CI = 1.45–1.68), *p* < 0.0001; T2DM: OR = 1.78 (95% CI = 1.60–1.97), *p* < 0.0001; HTN with T2DM: OR = 1.76 (95% CI = 1.59–1.95), *p* < 0.0001; dyslipidemia: OR = 1.08 (95% CI = 1.01–1.14), *p* = 0.0146; CVDs: OR = 1.12 (95% CI = 1.00–1.25), *p* = 0.0475). In the stratified analysis, high normal UACR was significantly associated with HTN, T2DM, HTN with T2DM, and dyslipidemia in subgroups.

**Conclusions:**

In summary, we observe a higher prevalence of HTN, T2DM, HTN with T2DM, dyslipidemia, and CVDs in abnormal UACR and reveal a significant association of UACR, even within the normal range, with HTN, T2DM, HTN with T2DM, dyslipidemia, and CVDs.

## Introduction

Albuminuria is a well-known indicator of renal damage, frequently a component of diabetic kidney disease (DKD) (1). Albuminuria in the nondiabetic or diabetic population is associated with an increased risk for adverse renal and cardiovascular events. Emerging data have shown that albuminuria is not only an initial manifestation of renal function loss but also a nonnegligible risk factor for cardiovascular diseases (CVDs), especially in populations with diabetes, hypertension (HTN), or dyslipidemia (2, 3). Therefore, albuminuria might possess great clinical value due to the ability to stratify risk for the general population.

Among diabetic adults in the United States, the prevalence of DKD is about 34.5%, and 16.8% present with albuminuria (4). The presence of albuminuria indicates a 2.5-fold increased risk of stroke, which is consistent with the findings delivered by Norfolk’s research (5, 6). Moreover, albuminuria, as measured by urine albumin to creatinine ratio (UACR), even within the normal range, is an effective predictor of HTN (7). Similarly, a 5-year-follow-up study conducted on Korean men demonstrated that high normal albuminuria (UACR <30 mg/g) could predict the increased risk of diabetes (8). A robust body of literature has also reported a strong, positive association between albuminuria and dyslipidemia in prediabetic and general population (9, 10). Hence, albuminuria might be valuable in clinical practice to identify the general population at high risk for CVDs, diabetes, HTN, and dyslipidemia.

Previous studies have described an association between albuminuria and related metabolic diseases. However, little literature focused on the prevalence of diabetes, HTN, diabetes with HTN and dyslipidemia in the Chinese population with different UACR levels. The present study is the first population-based study in the Chinese population and may uncover the incidence of diabetes, HTN, diabetes with HTN and dyslipidemia in individuals with different UACR levels. Therefore, this current study aimed to investigate the prevalence of diabetes, HTN, diabetes with HTN, CVDs, and dyslipidemia in different albuminuria ranges and explore the internal association between albuminuria and metabolic abnormalities.

## Methods and Materials

### Study Population and Design

**Figure 1:**
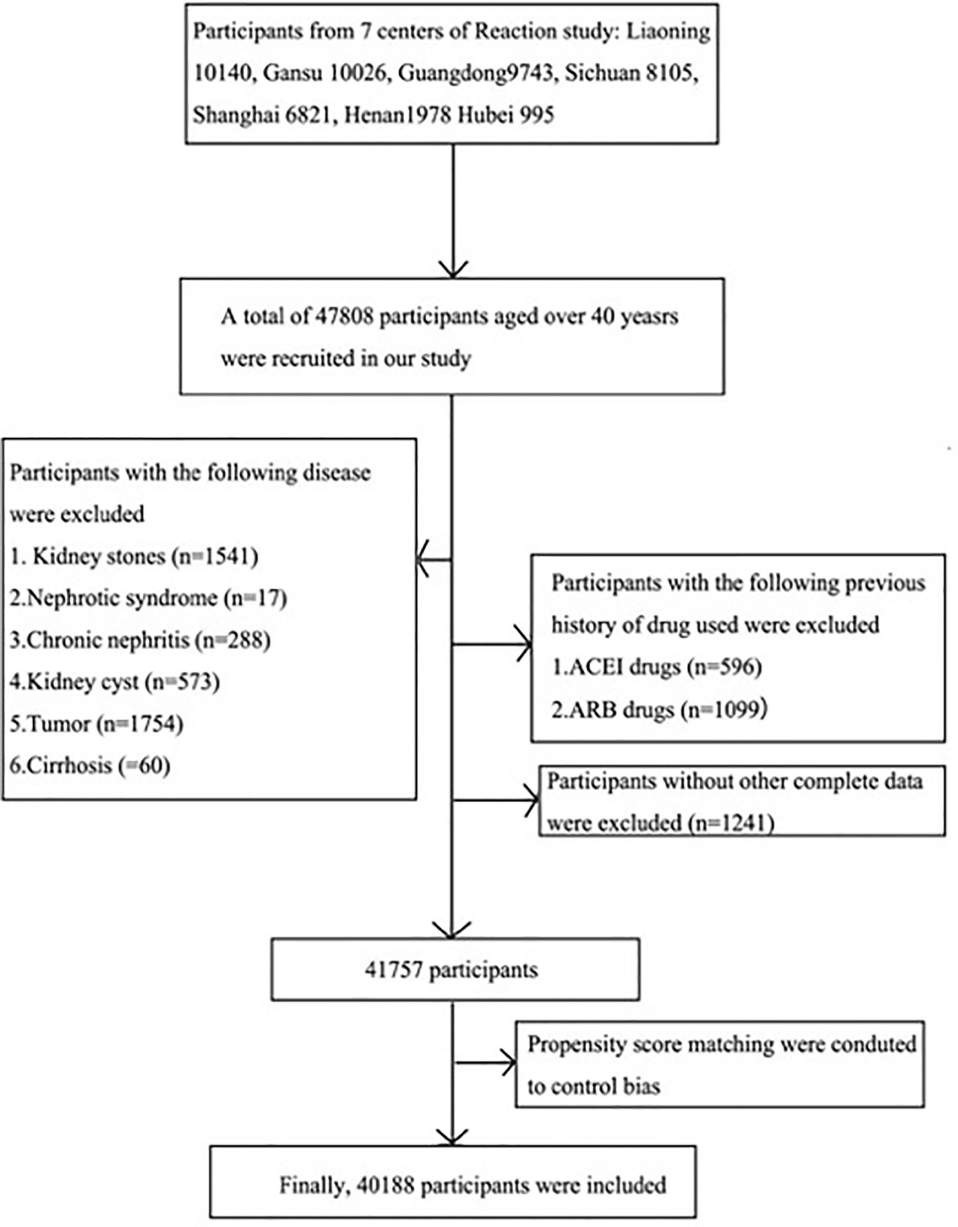
Flowchart of the selection of study participants.

The present study was drawn from the Risk Evaluation of Cancers in Chinese Diabetic Individuals (REACTION) study, which was conducted to investigate the association of diabetes and prediabetes with the risk of cancer in the Chinese population. Detailed information of the REACTION study has been described previously (11). The REACTION study was set up as a multicenter prospective observational study, and our study used baseline data from seven centers across China. A total of 47,808 participants aged over 40 years were recruited from May to December 2012 (Liaoning 10,140, Gansu 10,026, Guangzhou 9,743, Sichuan 8,105, Shanghai 6,821, Henan 1,978, Hubei 995). Participants diagnosed with kidney diseases, cancer, fatty liver, viral hepatitis, and cirrhosis, those using ACEI/ARB medicines, and those with missing data were excluded. A total of 41,757 participants were then enrolled. Given differences in the baseline characteristics between the two different UACR groups, the propensity score matching was performed to reduce potential bias. Ultimately, 40,188 eligible participants were included in this final analysis (**Figure 1**).

The staff received extensive training related to the study questionnaire and outcome measures before the investigation. The study was conducted following the Declaration of Helsinki, and the protocol was approved by the Clinical Research Ethics Committee of Rui-Jin Hospital Affiliated with the School of Medicine, Shanghai Jiao Tong University. Written informed consent was obtained from all participants before the study.

### Data Collection and Measurements

Data collection was performed by the well-trained staff, which included a standardized questionnaire, anthropometric measurements, blood collection, urine collection, and a 75-g oral glucose tolerance test (OGTT) or 100-g steamed-bread meal test. The self-administered questionnaire consisted of demographic information, the history of diabetes, HTN, dyslipidemia, kidney diseases, hepatic diseases, CVDs (coronary heart disease (CHD), myocardial infarction (MI), stroke), the current use of drugs (antihypertensive drugs, hypoglycemic drugs, and lipid-lowering drugs), and lifestyle including alcohol consumption and smoking consumption. Alcohol consumption was defined as follows: never; occasional drinkers who drank less than once a week; and regular drinkers who drank at least once a week for over 6 months. Smoking consumption was defined as follows: never; occasional smokers who smoked less than one cigarette per day or less than 7 cigarettes per week; and regular smokers who smoked at least one cigarette per day.

Anthropometric measurements, including height, weight, waist circumference (WC), and blood pressure, were performed by the same well‐trained staff. All participants were required to wear light clothing and take off shoes when weight and height were measured to the nearest 0.1 cm and 0.1 kg. WC was measured to the nearest 0.1 cm at the umbilical level when participants were in a standing position (12). Body mass index (BMI) was calculated using the following formula: BMI = body weight/height^2^ (kg/m^2^). Blood pressure and heart rate (HR) were recorded three times consecutively by the same well‐trained staff at 5-min intervals after participants were seated for at least 5 min at rest. The three measurements of blood pressure and HR were averaged for the final analysis.

Blood samples were collected by the experienced nurses in the morning after at least 12 h of overnight fasting. Participants with or without a history of diabetes underwent a 100-g steamed-bread meal test or 75-g OGTT, respectively. After 2 h, the second venous blood samples were obtained by the same well‐trained nurses. Fasting plasma glucose (FPG), 2 h postload blood glucose (PBG), hemoglobin A1c (HbA1c), serum triglycerides (TG), total cholesterol (TC), high-density lipoprotein cholesterol (HDL-C), low-density lipoprotein cholesterol (LDL-C), aspartate aminotransferase (AST), alanine aminotransferase (ALT), and creatinine (Cr) were measured at every center.

The estimated glomerular filtration rate (eGFR) was calculated based on the Chronic Kidney Disease Epidemiology Collaboration (CKD‐EPI) (13).

### Definition of Variables

Fasting midstream urine samples were collected in the morning to measure the urine albumin and creatinine concentration using chemiluminescence immunoassay in every center. UACR was calculated by dividing urine albumin in milligrams by urine creatinine in grams. The same range and units of UACR measurement were used in all seven centers. According to the KDIGO CKD guidelines, increased albuminuria was defined as UACR ≥30 mg/g, indicating kidney damage (14).

According to the WHO guidelines, type 2 diabetes mellitus (T2DM) was defined as FBG ≥7.0 mmol/L, or PBG ≥11.1 mmol/L, or diagnosed as T2DM by clinicians and meanwhile undergoing hypoglycemic-medication therapy. HTN was defined as systolic blood pressure (SBP) ≥140 mmHg or diastolic blood pressure (DBP) ≥90 mmHg, or diagnosed as HTN by clinicians and meanwhile undergoing antihypertensive-medication therapy. Dyslipidemia was defined as increased TC (≥6.20 mmol/L), LDL-C (>4.13 mmol/L), and TG (>2.25 mmol/L), decreased HDL-C (<1.03 mmol/L), or a combination of the above lipid abnormalities. HTN with T2DM was defined as a combination of HTN and T2DM. Stroke was defined as a self-reported history of language or physical dysfunction lasting over 24 h and ischemic or hemorrhagic stroke by clinical diagnosis. CHD events were defined as a self-report history of MI or angina or coronary revascularization by clinicians. CVDs were defined as a self-reported history of CHD, stroke, or MI events.

The information on CVDs was collected through an interviewer-assisted questionnaire. An open-ended question was asked: “Has a doctor or other health professional ever told you that you have myocardial infarction (MI), coronary heart disease (CHD), or stroke?” The self-reported history of CVDs in the questionnaire was validated in several communities. The related hospitalization records were reviewed by two physicians who were blind to the self-reported data.

Participants were divided into three groups according to their smoking frequency: no: never or have already quit smoking; occasional: smoking less than once a week or less than 7 cigarettes weekly; frequently: smoking one or more cigarettes daily for at least a half year. Similarly, participants were divided into three groups according to their alcohol intake frequency: no: never or have already quit drinking; occasional: drinking less than once a week; frequently: drinking more than once a week for at least a half year.

### Statistical Analysis

Empower(R) (www.empowerstats.com, X&Y Solutions Inc., Boston, MA) and R (http://www.Rproject.org) were used to perform the statistical analyses. Given differences in the baseline characteristics between eligible participants between the two groups of UACRs, propensity score matching was employed to control potential bias. Matching was performed using a 1:7 matching protocol to match all covariates, with a caliper width equal to 0.05 of the standard deviations (SD) of the logit of the propensity score.

Continuous variables with a nonnormal distribution were presented as median (Q1–Q3), and those with a normal distribution were presented as means ± SD. Categorical variables were expressed as *n*%. Differences in continuous variables were compared using the Kruskal–Wallis test, and when variables were categorical, the *χ* ^2^ test was used. Multivariate logistic regression analysis was performed to control potential confounding factors for identifying the associations of UACR with HTN, T2DM, HTN with T2DM, dyslipidemia, and CVDs in three models. Model 1 was unadjusted. Model 2 was adjusted for age and BMI. Model 3 was further adjusted for sex, SBP, DBP, HR, ALT, AST, eGFR, smoking habits, drinking habits, FBG, PBG, TC, TG, HDL-C, LDL-C, and history of medication, including antihypertensive drugs, hypoglycemic drugs, and lipid-lowering drugs. The odds ratio (OR) and corresponding 95% confidence intervals (95% CI) were calculated.

To thoroughly explore the associations between UACR and HTN, T2DM, HTN with T2DM, dyslipidemia, and CVDs, multivariate logistic regression analysis was also conducted in participants with normal albuminuria (UACR <30 mg/g). Moreover, to examine the internal link of the relationship between UACR and HTN, T2DM, HTN with T2DM, dyslipidemia, and CVDs, subgroups were stratified by the different history of HTN, T2DM, HTN with T2DM, dyslipidemia, and CVDs in the stratified analyses. All statistical tests were two-sided, and *p*-values <0.05 were considered statistically significant.

## Results

### Clinical Characteristics of the Study Population

After propensity score matching, a total of 40,188 participants with a median age (Q1–Q3) of 57.58 (52.40–63.93) years were included in this study, including 12,223 (30.41%) men and 27,965 (69.59%) women (**Table 1**). **Table 1** shows that 6,205 (15.44%) of participants had T2DM, 17,386 (43.26%) had HTN, 2,225 (5.54%) had CVDs, and 17,017 (42.34%) had dyslipidemia. Compared with normotensive participants, the hypertensive ones were older; had a larger BMI, ALT, AST, SBP, DBP, HR, TC, TG, LDL-C, FBG, PBG, HbA1c, and UACR; lower eGFR; were less frequent smokers; more frequent drinkers; and a higher prevalence of T2DM, HTN with T2DM, CVDs, and dyslipidemia (**Supplementary Table S1**). Compared with non-T2DM participants, the diabetic ones were older; had a larger BMI, ALT, SBP, DBP, HR, TG, FBG, PBG, HbA1c, and UACR; lower eGFR, LDL-C, and HDL-C; were more frequent smokers and drinkers; and a higher prevalence of HTN, HTN with T2DM, CVDs, and dyslipidemia. Similar results were found in HTN with T2DM. Similarly, participants with dyslipidemia had an inferior control of blood pressure, glucose, and lipid; a higher prevalence of HTN, T2DM, and CVDs; and were more frequent smokers or drinkers (**Supplementary Tables S1**–**S4**).

**Table 1:** Characteristics of total population.

Variables	All participants
Age	57.58 (52.40–63.93)
BMI	24.34 (22.19–26.64)
ALT	15.00 (11.00–21.00)
AST	20.00 (17.00–25.00)
SBP	130.00 (117.00–145.00)
DBP	77.00 (70.00–84.00)
HR	78.00 (71.00–86.00)
TC	5.06 (4.33–5.79)
TG	1.38 (0.98–1.99)
LDL-C	2.94 (2.36–3.55)
HDL-C	1.29 (1.09–1.52)
FBG	5.54 (5.11–6.19)
PBG	7.41 (6.02–9.78)
HbA1c	5.90 (5.60–6.30)
eGFR	95.32 (90.93–99.01)
UACR	10.17 (5.92–20.13)
Sex	*N* (%)
Men	12,223 (30.41%)
Women	27,965 (69.59%)
Smoking	
No	34,287 (85.32%)
Occasional	1,209 (3.01%)
Frequently	4,692 (11.68%)
Drinking	
No	30,136 (74.99%)
Occasional	7,457 (18.56%)
Frequently	2,595 (6.46%)
Antihypertensive drugs	
Yes	6,452 (16.05%)
No	3,3736 (83.95%)
Hypoglycemic drugs	
Yes	3,776 (9.40%)
No	36,412 (90.60%)
Lipid-lowering drugs	
Yes	421 (1.05%)
No	39,767 (98.95%)
T2DM	
Yes	6,205 (15.44%)
No	33,983 (84.56%)
HTN	
Yes	17,386 (43.26%)
No	22,802 (56.74%)
CVDs	
Yes	2,225 (5.54%)
No	37,963 (94.46%)
Dyslipidemia	
Yes	17,017 (42.34%)
No	23,171 (57.66%)

Data were mean ± SD or median (Q1–Q3) for nonnormal distribution of variables or numbers (%) for categorical variables.

BMI, body mass index; SBP, systolic blood pressure; DBP, diastolic blood pressure; ALT, alanine transferase; AST, aspartate transferase; HR, heart rate; TG, triglyceride; TC, high cholesterol; LDL-C, low-density lipoprotein cholesterol; HDL-C, high-density lipoprotein cholesterol; FBG, fasting plasma glucose; PBG, 2 h postload blood glucose; HbA1c, glycosylated hemoglobin; eGFR, estimated glomerular filtration rate; T2DM, type 2 diabetes mellitus; CVDs, cardiovascular diseases; UACR, urinary albumin to creatinine ratio.

### Associations of UACR With HTN, T2DM, HTN With T2DM, Dyslipidemia, and CVDs in the Total Population

Multiple logistic regression models that consider the association of albuminuria with the individual prevalence of HTN, T2DM, HTN with T2DM, dyslipidemia, and CVDs were constructed. **Table 2** shows OR and 95% CI of HTN, T2DM, HTN with T2DM, dyslipidemia, CVDs with continuous UACR, and categories of UACR in the total population of three different models. As seen in **Table 2**, UACR is significantly associated with HTN, T2DM, HTN with T2DM, dyslipidemia, and CVDs in models 1 and 2. Even after further adjustments in model 3, UACR remained significantly associated with HTN, T2DM, HTN with T2DM, dyslipidemia, and CVDs, However, in model 3, the adjusted OR of UACR (≥30 mg/g) was not as remarkable as in model 2, but the association of UACR with T2DM was most significant in model 3 (HTN: OR = 1.89 (95% CI = 1.77–2.01), *p* < 0.0001 in model 2 vs. OR = 1.56 (95% CI = 1.45–1.68), *p* < 0.0001 in model 3; T2DM: OR = 2.21 (95% CI = 2.06–2.36), *p* < 0.0001 in model 2 vs. OR = 1.78 (95% CI = 1.60–1.97), *p* < 0.0001 in model 3; HTN with T2DM: OR = 2.50 (95% CI = 2.31–2.70), *p* < 0.0001 in model 2 vs. OR = 1.76 (95% CI = 1.59–1.95), *p* < 0.0001 in model 3; dyslipidemia: OR = 1.18 (95% CI = 1.12–1.25), *p* < 0.0001 in model 2 vs. OR = 1.08 (95% CI = 1.01–1.14), *p* = 0.0146 in model 3; CVDs: OR = 1.30 (95% CI = 1.17–1.45), *p* < 0.0001 in model 2 vs. OR = 1.12 (95% CI = 1.00–1.25), *p* = 0.0475 in model 3), indicating the blood glucose, pressure, and lipid level possessing the effects of the metabolic diseases. The association between continuous values of UACR is also shown in **Table 2**, which was consistent with the findings delivered by categorical values.

**Table 2:** Associations of UACR with HTN, T2DM, HTN with T2DM, dyslipidemia, and CVDs in the general population.

Exposure	Model 1^a^		Model 2^b^		Model 3^c^	
	OR (95% CI)	*p*-value	OR (95% CI)	*p*-value	OR (95% CI)	*p*-value
HTN						
Continuous UACR	1.00 (1.00, 1.00)	<0.0001	1.00 (1.00, 1.00)	0.0001	1.00 (1.00, 1.00)	0.0004
Categorical UACR						
<30 mg/g	1.0		1.0		1.0	
≥30 mg/g	2.33 (2.20, 2.47)	<0.0001	1.89 (1.77, 2.01)	<0.0001	1.56 (1.45, 1.68)	<0.0001
T2DM						
Continuous UACR	1.00 (1.00, 1.00)	0.0017	1.00 (1.00, 1.00)	0.0018	1.00 (1.00, 1.00)	0.0106
Categorical UACR						
<30 mg/g	1.0		1.0		1.0	
≥30 mg/g	2.58 (2.42, 2.76)	<0.0001	2.21 (2.06, 2.36)	<0.0001	1.78 (1.60, 1.97)	<0.0001
HTN with T2DM						
Continuous UACR	1.00 (1.00, 1.00)	0.0002	1.00 (1.00, 1.00)	0.0001	1.00 (1.00, 1.00)	<0.0001
Categorical UACR						
<30 mg/g	1.0		1.0		1.0	
≥30 mg/g	3.09 (2.87, 3.33)	<0.0001	2.50 (2.31, 2.70)	<0.0001	1.76 (1.59, 1.95)	<0.0001
Dyslipidemia						
Continuous UACR	1.00 (1.00, 1.00)	0.1352	1.00 (1.00, 1.00)	0.2151	1.00 (1.00, 1.00)	0.4618
Categorical UACR						
<30 mg/g	1.0		1.0		1.0	
≥30 mg/g	1.28 (1.21, 1.35)	<0.0001	1.18 (1.12, 1.25)	<0.0001	1.08 (1.01, 1.14)	0.0146
CVDs						
Continuous UACR	1.00 (1.00, 1.00)	0.4427	1.00 (1.00, 1.00)	0.7463	1.00 (1.00, 1.00)	0.8393
Categorical UACR						
<30 mg/g	1.0		1.0		1.0	
≥30 mg/g	1.86 (1.68, 2.06)	<0.0001	1.30 (1.17, 1.45)	<0.0001	1.12 (1.00, 1.25)	0.0475

a Unadjusted.

b Adjusted for age and BMI.

c Additionally adjusted for sex, ALT, AST, eGFR, SBP, DBP, HR, TG, TC, LDL, HDL, FBG, PBG, smoking, drinking, antihypertensive drugs, hypoglycemic drugs, and lipid-lowering drugs based on model 2.

OR, odds ratio; CI, confidential interval; BMI, body mass index; ALT, alanine transferase; AST, aspartate transferase; eGFR, estimated glomerular filtration rate; SBP, systolic blood pressure; DBP, diastolic blood pressure; HR, heart rate; TG, triglyceride; TC, high cholesterol; LDL-C, low-density lipoprotein cholesterol; HDL-C, high-density lipoprotein cholesterol; FBG, fasting plasma glucose; PBG, 2 h postload blood glucose; T2DM, type 2 diabetes mellitus; CVDs, cardiovascular diseases; UACR, urinary albumin to creatinine ratio.

### Associations of UACR With HTN, T2DM, HTN With T2DM, Dyslipidemia and CVDs in Participants With Normal UACR (<30 mg/g)

To thoroughly explore the association of albuminuria with such diseases, multiple logistic regression models were also constructed in participants with the normal range of albuminuria (UACR <30 mg/g) as shown in **Table 3**. These results indicate that compared with participants with lower UACR levels, participants with higher normal UACR levels (HTN: the third quintile to the fifth quintile; T2DM: the third quintile to the fifth quintile; HTN with T2DM: the second quintile to the fifth quintile; dyslipidemia: the third quintile to the fifth quintile; CVDs: the fifth quintile) were also significantly associated with HTN, T2DM, HTN with T2DM, dyslipidemia, and CVDs even after adjustments for confounding factors in model 3. In model 3, the ORs for HTN was increased significantly from the second quintile, with ORs 1.10 (1.02, 1.19) for quintile 3, 1.20 (1.11, 1.29) for quintile 4, and 1.28 (1.14, 1.44) for quintile 5. Similar results and tendency were observed in T2DM, HTN with T2DM, and dyslipidemia (T2DM: Q3: OR = 1.33 (95% CI = 1.15–1.54), *p* = 0.0001; Q4: OR = 1.57 (95% CI = 1.36–1.82), *p* < 0.0001; Q5: OR = 2.00 (95% CI = 1.65–2.44), *p* < 0.0001; HTN with T2DM: Q2 : OR = 1.20 (95% CI = 1.02–1.42), *p* = 0.0303, Q3: OR = 1.19 (95% CI = 1.01–1.41), *p* = 0.0329; Q4: OR = 1.43 (95% CI = 1.22–1.68), *p* < 0.0001; Q5: OR = 1.54 (95% CI = 1.25–1.90), *p* < 0.0001; dyslipidemia: Q3: OR = 1.12 (95% CI = 1.05–1.19), *p* = 0.0008; Q4: OR = 1.12 (95% CI = 1.04–1.19), *p* = 0.0010; Q5: OR = 1.14 (95% CI = 1.03–1.26), *p* = 0.0088; CVDs: Q5: OR = 1.23 (95% CI = 1.00–1.51), *p* = 0.0482). Similar positive associations were detected in participants with the normal range of albuminuria when UACR was a continuous variable (HTN: OR = 1.01 (95% CI = 1.01–1.02), *p* < 0.0001; T2DM: OR = 1.03 (95% CI = 1.02–1.04), *p* < 0.0001; HTN with T2DM: OR = 1.02 (95% CI = 1.01–1.03), *p* < 0.0001; dyslipidemia: OR = 1.01 (95% CI = 1.00–1.01), *p* = 0.0005; CVDs: OR = 1.00 (95% CI = 0.99–1.01), *p* = 0.8208).

**Table 3:** Associations of UACR with HTN, T2DM, HTN with T2DM, dyslipidemia, and CVDs in participants with normal UACR (<30 mg/g).

Exposure	Model 1^a^		Model 2^b^		Model 3^c^	
	OR (95% CI)	*p*-value	OR (95% CI)	*p*-value	OR (95% CI)	*p*-value
HTN						
Continuous UACR	1.03 (1.03, 1.03)	<0.0001	1.02 (1.02, 1.03)	<0.0001	1.01 (1.01, 1.02)	<0.0001
UACR quintiles						
Q1	1.0		1.0		1.0	
Q2	1.11 (1.04, 1.19)	0.0012	1.08 (1.01, 1.16)	0.0218	0.99 (0.92, 1.07)	0.8029
Q3	1.34 (1.25, 1.43)	<0.0001	1.28 (1.19, 1.37)	<0.0001	1.10 (1.02, 1.19)	0.0123
Q4	1.65 (1.55, 1.76)	<0.0001	1.44 (1.34, 1.54)	<0.0001	1.20 (1.11, 1.29)	<0.0001
Q5	1.80 (1.64, 1.98)	<0.0001	1.53 (1.38, 1.70)	<0.0001	1.28 (1.14, 1.44)	<0.0001
T2DM						
Continuous UACR	1.04 (1.03, 1.04)	<0.0001	1.03 (1.03, 1.04)	<0.0001	1.03 (1.02, 1.04)	<0.0001
UACR quintiles						
Q1	1.0		1.0		1.0	
Q2	1.21 (1.09, 1.34)	0.0003	1.18 (1.07, 1.31)	0.0013	1.14 (0.99, 1.33)	0.0776
Q3	1.42 (1.29, 1.57)	<0.0001	1.35 (1.23, 1.50)	<0.0001	1.33 (1.15, 1.54)	0.0001
Q4	1.89 (1.72, 2.07)	<0.0001	1.69 (1.53, 1.86)	<0.0001	1.57 (1.36, 1.82)	<0.0001
Q5	2.41 (2.13, 2.74)	<0.0001	2.14 (1.88, 2.44)	<0.0001	2.00 (1.65, 2.44)	<0.0001
HTN with T2DM						
Continuous UACR	1.04 (1.04, 1.05)	<0.0001	1.04 (1.03, 1.04)	<0.0001	1.02 (1.01, 1.03)	<0.0001
UACR quintiles						
Q1	1.0		1.0		1.0	
Q2	1.34 (1.17, 1.53)	<0.0001	1.30 (1.14, 1.49)	0.0002	1.20 (1.02, 1.42)	0.0303
Q3	1.57 (1.38, 1.79)	<0.0001	1.44 (1.26, 1.65)	<0.0001	1.19 (1.01, 1.41)	0.0329
Q4	2.26 (2.00, 2.55)	<0.0001	1.91 (1.69, 2.17)	<0.0001	1.43 (1.22, 1.68)	<0.0001
Q5	2.72 (2.32, 3.19)	<0.0001	2.28 (1.93, 2.68)	<0.0001	1.54 (1.25, 1.90)	<0.0001
Dyslipidemia						
Continuous UACR	1.01 (1.01, 1.01)	<0.0001	1.01 (1.00, 1.01)	<0.0001	1.01 (1.00, 1.01)0.0005	0.0005
UACR quintiles						
Q1	1.0		1.0		1.0	
Q2	1.02 (0.96, 1.09)	0.4546	1.03 (0.96, 1.09)	0.4324	1.02 (0.96, 1.09)	0.5522
Q3	1.13 (1.06, 1.20)	0.0001	1.13 (1.06, 1.20)	0.0002	1.12 (1.05, 1.19)	0.0008
Q4	1.17 (1.10, 1.25)	<0.0001	1.14 (1.07, 1.21)	<0.0001	1.12 (1.05, 1.19)	0.0010
Q5	1.21 (1.10, 1.33)	<0.0001	1.17 (1.06, 1.28)	0.0017	1.14 (1.03, 1.26)	0.0088
CVDs						
Continuous UACR	1.02 (1.02, 1.03)	<0.0001	1.01 (1.00, 1.02)	0.0063	1.00 (0.99, 1.01)	0.8208
UACR quintiles						
Q1	1.0		1.0		1.0	
Q2	1.08 (0.92, 1.26)	0.3428	1.00 (0.86, 1.17)	0.9811	1.00 (0.85, 1.17)	0.9908
Q3	1.28 (1.11, 1.49)	0.0010	1.10 (0.95, 1.28)	0.2117	1.10 (0.94, 1.28)	0.2477
Q4	1.49 (1.29, 1.72)	<0.0001	1.13 (0.97, 1.31)	0.1178	1.09 (0.93, 1.27)	0.2827
Q5	1.82 (1.50, 2.21)	<0.0001	1.34 (1.10, 1.64)	0.0042	1.23 (1.00, 1.51)	0.0482

a Unadjusted.

b Adjusted for age and BMI.

c Additionally adjusted for sex, ALT, AST, eGFR, SBP, DBP, HR, TG,TC, LDL, HDL, FBG, PBG, smoking, drinking, antihypertensive drugs, hypoglycemic drugs, and lipid-lowering drugs based on model 2.

OR, odds ratio; CI, confidential interval; BMI, body mass index; ALT, alanine transferase; AST, aspartate transferase; eGFR, estimated glomerular filtration rate; SBP, systolic blood pressure; DBP, diastolic blood pressure; HR, heart rate; TG, triglyceride; TC, high cholesterol; LDL-C, low-density lipoprotein cholesterol; HDL-C, high-density lipoprotein cholesterol; FBG, fasting plasma glucose; PBG, 2 h postload blood glucose; T2DM, type 2 diabetes mellitus; CVDs, cardiovascular diseases; UACR, urinary albumin to creatinine ratio.

### Stratified Analysis of Associations Between UACR and HTN, T2DM, HTN With T2DM, Dyslipidemia, and CVDs in Participants With Normal UACR (<30 mg/g)

Stratified analyses were conducted in the different subgroups of HTN, T2DM, dyslipidemia, and CVDs to validate the abovementioned results, as shown in **Table 4**. The present study found that compared with lower UACR, higher normal UACR (the third, fourth, and fifth quintiles) was closely associated with HTN in both subgroups of HTN, T2DM, and dyslipidemia and the subgroup without CVDs. To be noted, these associations were the most significant in participants that were both in the subgroup of the fifth quintile of UACR and the subgroups of normal blood glucose (OR = 1.64 (95% CI = 1.46–1.84), *p* < 0.0001), dyslipidemia (OR = 1.32 (95% CI = 1.11–1.57), *p* = 0.0018), and CVD-free (OR = 1.29 (95% CI = 1.14–1.45), *p* < 0.0001). Similarly, the most significant association of UACR with T2DM were both in the fifth quintile of UACR subgroup and normal blood pressure (OR = 2.08 (95% CI = 1.55–2.78), *p* < 0.0001), dyslipidemia (OR = 2.01 (95% CI = 1.54–2.62), *p* < 0.0001), and CVD-free (OR = 2.05 95%CI 1.68-2.51), *p* < 0.0001) subgroups. The association between the fifth quintile of UACR and HTN with T2DM was more significant in dyslipidemia (OR = 1.86 (95% CI = 1.41-2.47), *p* < 0.0001) and CVD-free (OR = 1.59 (95% CI = 1.28-1.99), *p* < 0.0001) subgroups. The most significant association of the fifth quintile of UACR with dyslipidemia was detected in participants without HTN (OR = 1.15 (95% CI = 1.01–1.32), *p* = 0.0418) and without T2DM and CVDs (T2DM: OR = 1.13 95%CI 1.02–1.27), *p* = 0.0248; CVDs: OR = 1.14 95%CI 1.03–1.26), *p* = 0.0124). However, no significant association between the fifth quintile of UACR and CVDs was observed in subgroups of HTN, T2DM, and dyslipidemia.

**Table 4:** Stratified analysis of associations between UACR and HTN, T2DM, HTN with T2DM, dyslipidemia, and CVDs in participants with normal UACR (<30 mg/g) in model 3.

Exposure	HTN		T2DM		Dyslipidemia		CVDs	
	OR (95% CI); *p*-value		OR (95% CI); *p*-value		OR (95% CI); *p*-value		OR (95% CI); *p*-value	
	No	Yes	No	Yes	No	Yes	No	Yes
HTN								
Continuous UACR			1.02 (1.02, 1.03); <0.0001	1.02 (1.01, 1.03); 0.0001	1.01 (1.01, 1.02); <0.0001	1.01 (1.01, 1.02); <0.0001	1.01 (1.01, 1.02); <0.0001	1.01 (0.99, 1.03); 0.2995
UACR quintiles								
Q1			1.0	1.0	1.0	1.0	1.0	1.0
Q2			1.06 (0.98, 1.14); 0.1329	1.32 (1.07, 1.62); 0.0089	1.01 (0.91, 1.11); 0.8782	0.96 (0.86, 1.08); 0.5048	1.00 (0.93, 1.09); 0.9179	0.71 (0.49, 1.03); 0.0723
Q3			1.31 (1.22, 1.42); <0.0001	1.29 (1.06, 1.58); 0.0116	1.11 (1.00, 1.23); 0.0467	1.08 (0.96, 1.21); 0.2051	1.11 (1.02, 1.20); 0.0103	0.97 (0.67, 1.39); 0.8530
Q4			1.46 (1.35, 1.58); <0.0001	1.57 (1.30, 1.91); <0.0001	1.19 (1.07, 1.32); 0.0013	1.19 (1.06, 1.34); 0.0031	1.21 (1.12, 1.31); <0.0001	0.97 (0.67, 1.41); 0.8861
Q5			1.64 (1.46, 1.84); <0.0001	1.45 (1.13, 1.87); 0.0037	1.23 (1.05, 1.44); 0.0099	1.32 (1.11, 1.57); 0.0018	1.29 (1.14, 1.45); <0.0001	1.13 (0.67, 1.90); 0.6394
T2DM								
Continuous UACR	1.04 (1.03, 1.05); <0.0001	1.03 (1.02, 1.04); <0.0001			1.02 (1.01, 1.04); <0.0001	1.03 (1.02, 1.04); <0.0001	1.03 (1.03, 1.04); <0.0001	1.04 (1.01, 1.06); 0.0082
UACR quintiles								
Q1	1.0	1.0			1.0	1.0	1.0	1.0
Q2	0.95 (0.77, 1.18); 0.6589	1.36 (1.10, 1.68); 0.0045			1.19 (0.95, 1.48); 0.1216	1.09 (0.89, 1.34); 0.4066	1.15 (0.99, 1.34); 0.0711	0.99 (0.49, 2.02); 0.9838
Q3	1.31 (1.07, 1.61); 0.0105	1.40 (1.13, 1.72); 0.0019			1.32 (1.06, 1.64); 0.0136	1.30 (1.07, 1.59); 0.0085	1.33 (1.14, 1.55); 0.0002	1.37 (0.71, 2.64); 0.3463
Q4	1.54 (1.25, 1.90); <0.0001	1.67 (1.36, 2.05); <0.0001			1.42 (1.14, 1.78); 0.0017	1.64 (1.35, 1.98); <0.0001	1.53 (1.32, 1.78); <0.0001	2.35 (1.27, 4.36); 0.0068
Q5	2.08 (1.55, 2.78); <0.0001	2.02 (1.55, 2.65); <0.0001			1.88 (1.40, 2.53); <0.0001	2.01 (1.54, 2.62); <0.0001	2.05 (1.68, 2.51); <0.0001	1.51 (0.65, 3.49); 0.3381
HTN with T2DM								
Continuous UACR					1.01 (1.00, 1.02); 0.1233	1.02 (1.02, 1.03); <0.0001	1.02 (1.01, 1.03); <0.0001	1.02 (1.00, 1.04); 0.1167
UACR quintiles								
Q1					1.0	1.0	1.0	1.0
Q2					1.15 (0.91, 1.47); 0.2468	1.23 (0.98, 1.54); 0.0731	1.24 (1.04, 1.47); 0.0142	0.76 (0.42, 1.39); 0.3741
Q3					1.19 (0.93, 1.51); 0.1666	1.18 (0.95, 1.48); 0.1342	1.21 (1.02, 1.44); 0.0263	0.97 (0.54, 1.71); 0.9047
Q4					1.26 (0.99, 1.60); 0.0551	1.54 (1.25, 1.91); <0.0001	1.42 (1.20, 1.68); <0.0001	1.50 (0.88, 2.56); 0.1382
Q5					1.16 (0.84, 1.60); 0.3757	1.86 (1.41, 2.47); <0.0001	1.59 (1.28, 1.99); <0.0001	1.06 (0.52, 2.15); 0.8657
Dyslipidemia								
Continuous UACR	1.01 (1.00, 1.01); 0.0030	1.01 (1.00, 1.01); 0.0346	1.01 (1.00, 1.01); 0.0051	1.01 (1.00, 1.02); 0.0678			1.01 (1.00, 1.01); 0.0012	1.01 (1.00, 1.03); 0.0965
UACR quintiles								
Q1	1.0	1.0	1.0	1.0			1.0	1.0
Q2	1.06 (0.98, 1.15); 0.1493	0.96 (0.87, 1.07); 0.4932	1.05 (0.98, 1.12); 0.1873	0.80 (0.66, 0.98); 0.0288			1.02 (0.95, 1.09); 0.6267	1.11 (0.81, 1.52); 0.4998
Q3	1.17 (1.08, 1.27); 0.0003	1.05 (0.95, 1.17); 0.3556	1.12 (1.05, 1.20); 0.0014	1.04 (0.86, 1.26); 0.7029			1.11 (1.03, 1.18); 0.0034	1.41 (1.04, 1.91); 0.0265
Q4	1.15 (1.05, 1.26); 0.0016	1.07 (0.97, 1.19); 0.1718	1.12 (1.04, 1.20); 0.0028	1.04 (0.87, 1.25); 0.6728			1.11 (1.04, 1.19); 0.0021	1.30 (0.96, 1.75); 0.0919
Q5	1.15 (1.01, 1.32); 0.0418	1.12 (0.97, 1.30); 0.1153	1.13 (1.02, 1.26); 0.0248	1.09 (0.86, 1.39); 0.4576			1.14 (1.03, 1.26); 0.0124	1.23 (0.83, 1.83); 0.3082
CVDs								
Continuous UACR	0.99 (0.98, 1.00); 0.1452	1.00 (0.99, 1.01); 0.8282	0.99 (0.99, 1.00); 0.2259	1.00 (0.99, 1.01); 0.9338	1.02 (0.98, 1.06); 0.4502	1.00 (0.99, 1.00); 0.2564		
UACR quintiles								
Q1	1.0	1.0	1.0	1.0	1.0	1.0		
Q2	1.15 (0.89, 1.48); 0.2779	0.83 (0.67, 1.02); 0.0805	0.91 (0.76, 1.08); 0.2807	0.91 (0.76, 1.08); 0.2807	1.29 (0.56, 2.95); 0.5480	0.93 (0.79, 1.09); 0.3648		
Q3	1.01 (0.78, 1.31); 0.9308	0.91 (0.75, 1.11); 0.3622	0.94 (0.79, 1.13); 0.5255	0.99 (0.70, 1.40); 0.9446	1.07 (0.46, 2.52); 0.8709	0.94 (0.80, 1.11); 0.4885		
Q4	0.89 (0.68, 1.16); 0.3915	0.88 (0.73, 1.07); 0.2117	0.83 (0.69, 0.99); 0.0377	1.12 (0.81, 1.55) 0.4966	1.76 (0.76, 4.07); 0.1887	0.88 (0.75, 1.03); 0.1171		
Q5	0.91 (0.61, 1.35); 0.6380	1.01 (0.79, 1.30); 0.9290	1.03 (0.81, 1.32); 0.797	0.95 (0.63, 1.44); 0.8127	1.06 (0.32, 3.51); 0.9289	0.98 (0.79, 1.21); 0.8569		

Model 1: unadjusted. Model 2: adjusted for age and BMI. Model 3: additionally adjusted for sex, ALT, AST, eGFR, SBP, DBP, HR, TG,TC, LDL,HDL, FBG, PBG, smoking, drinking, antihypertensive drugs, hypoglycemic drugs, and lipid-lowering drugs based on model 2.

OR, odds ratio; CI, confidential interval; BMI, body mass index; ALT, alanine transferase; AST, aspartate transferase; eGFR, estimated glomerular filtration rate; SBP, systolic blood pressure; DBP, diastolic blood pressure; HR, heart rate; TG, triglyceride; TC, high cholesterol; LDL-C, low-density lipoprotein cholesterol; HDL-C, high-density lipoprotein cholesterol; FBG, fasting plasma glucose; PBG, 2 h postload blood glucose; T2DM, type 2 diabetes mellitus; CVDs, cardiovascular diseases; UACR, urinary albumin to creatinine ratio.

## Discussion

**Figure 2:**
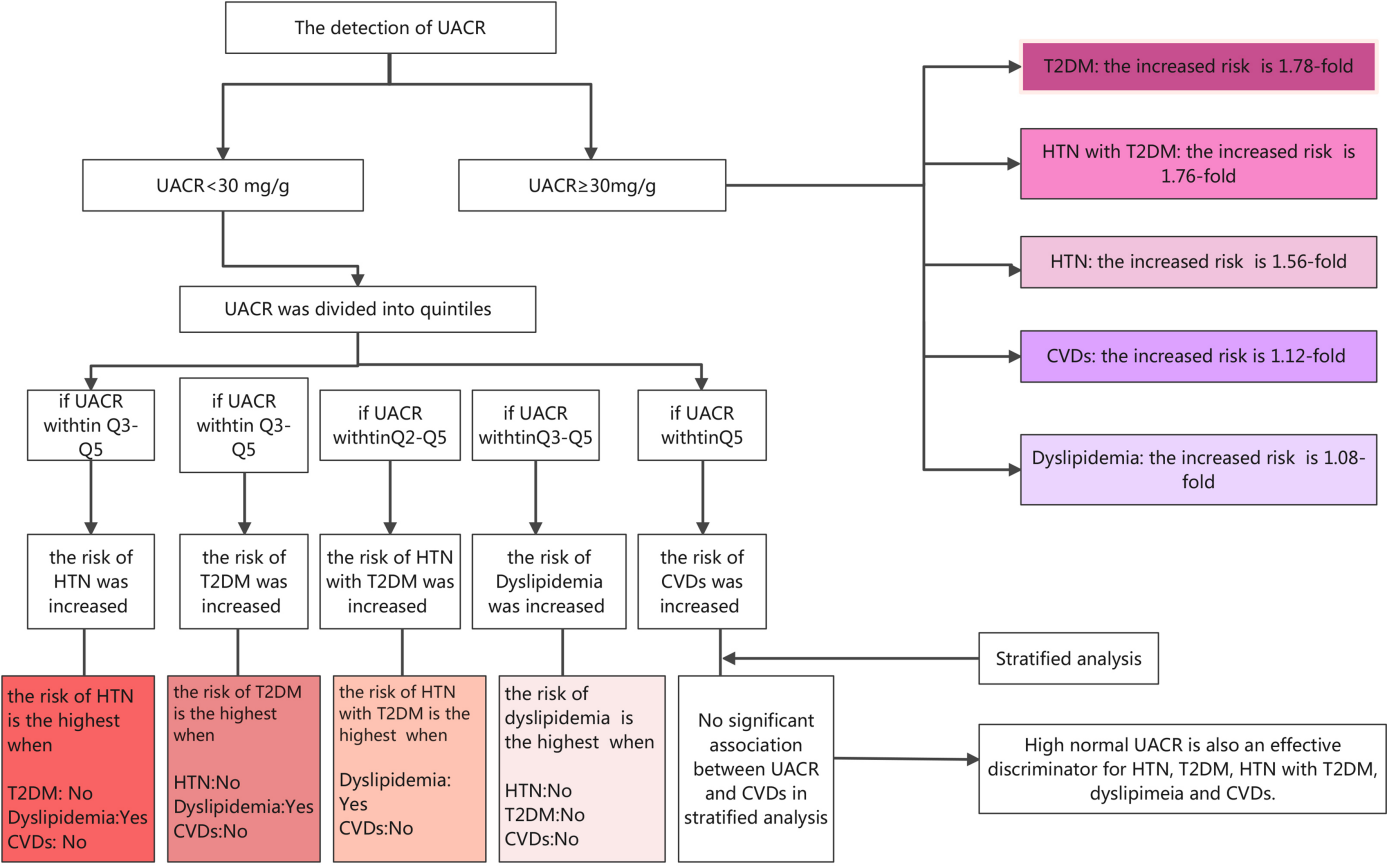
The summary of the conclusion of this study.

As far as we all know, this is the first study conducted on the Chinese population to observe the prevalence of HTN, T2DM, HTN with T2DM, dyslipidemia, and CVDs with different UACR levels and explore the associations between albuminuria and the above diseases, even when albuminuria within the normal range. The following are the main findings of this current study, as shown in **Figure 2**: (1) compared with participants with normal albuminuria (UACR <30 mg/g), participants with abnormal albuminuria (UACR ≥30 mg/g) had a higher prevalence of HTN (40.26% vs. 61.11%), T2DM (13.27% vs. 28.34%), HTN with T2DM (7.79% vs. 20.71%), dyslipidemia (41.48% vs. 47.49%), and CVDs (4.98% vs. 8.88%), indicating the close relationship between albuminuria and metabolic diseases. (2) UACR is significantly associated with HTN, T2DM, HTN with T2DM, dyslipidemia, and CVDs after adjusting for a broad spectrum of confounding factors. (3) High normal UACR is also associated with HTN, T2DM, HTN with T2DM, dyslipidemia, and CVDs, and the association between high normal UACR and T2DM is the most significant. (4) Further stratification shows that when UACR is at the fifth quintile, participants without diabetes and CVDs and with dyslipidemia have higher risks of HTN; those without HTN and CVDs and with dyslipidemia have higher risks of T2DM; those with dyslipidemia and without CVDs have higher risks of HTN with T2DM; those without HTN, T2DM, and CVDs have higher risks of dyslipidemia. Therefore, UACR is an effective discriminator for the risk of HTN, T2DM, HTN with T2DM, dyslipidemia, and CVDs even when within the normal range. It is not only people with abnormal UACR but that those with high normal UACR should be vigilant in the detection, prevention, and control of blood glucose, pressure, and lipids to prevent and reduce the risk of HTN, T2DM, HTN with T2DM, dyslipidemia, and CVDs.

It is widely accepted that HTN is an important risk factor contributing to mortality worldwide, and albuminuria plays a crucial role in the initiation and progression of HTN in previous studies (15, 16). This research has described that albuminuria excretion of more than 6 mg/day can effectively predict the progression of HTN. Notably, a growing number of studies in the western population have pointed out that a significant association between albuminuria and HTN was not only restricted to abnormal albuminuria (UACR ≥30 mg/g) but also albuminuria below the normal threshold (UACR <30 mg/g) was found associated with HTN. The Framingham Heart Study has reported that men with UACR >6.66 mg/g and women with >15.24 mg/g had an approximately 2-fold risk of developing HTN, suggesting that UACR is a useful biomarker for identifying individuals at high risk for HTN (17). Furthermore, in postmenopausal women without diabetes, there was a positive association between UACR within the normal range and HTN, suggesting a reassessment of normal albuminuria excretion (18).

Similarly, in our study, we found that not only abnormal UACR but an increase in UACR even within the normal range is strongly associated with HTN, especially in people without T2DM and CVDs. Although the prevalence of HTN was higher in people with abnormal UACR than those with normal UACR (61.11% vs. 40.26%), people with high normal UACR (the fifth quintile) have a 1.28-fold risk of HTN than those with low UACR (the first quintile) within the normal range in our study (**Table 3**). Our results clearly showed that high normal UACR is closely associated with HTN, and the association is independent of eGFR levels, which were consistent with previous studies. Systemic and glomerular vascular abnormalities were potential physiologic links between albuminuria and HTN (19, 20). Albuminuria could be caused by physiologic abnormalities of glomerular endothelial cells, the glomerular basement membrane, and podocytes, leading to increased albumin filtration. Increased albuminuria likely reflects generalized microvascular endothelial cell damage (21), which possibly predisposes to an increased atherogenic lipoprotein accumulation within the subendothelial cell space (22). A cohort study based on the Japanese population, which followed 412 normotensive individuals without diabetes for a median of 6.7 years, observed that a slight increase in UACR was closely associated with the incidence of HTN. Moreover, this study pointed out that UACR is a predictor of increased blood pressure and incident HTN (23), suggesting that increased UACR is partly due to increased blood pressure below the level of diagnosis of HTN.

Albuminuria is closely associated not only with HTN but also with T2DM. There is strong evidence that albuminuria could be well indicative of microvascular dysfunction (21). Compared with individuals without T2DM, individuals with T2DM have markedly impaired microvascular function (24, 25). Louis et al. pointed out that albuminuria levels were independently associated with the severity of cardiac macrovascular function in individuals with T2DM (26). Although among diabetic individuals with normal ventricular diastolic function, the prevalence of cardiac macrovascular dysfunction was higher, especially in those with abnormal albuminuria, which was in line with our results. UACR is significantly higher in individuals with T2DM than those without T2DM in the present research (**Table 1**), indicating a close link between UACR and T2DM. It was well confirmed that UACR is not only a known indicator of kidney damage but also an effective predictor of atherogenic state. Accordingly, the results of population-based studies supported that UACR is valuable in predicting cardiovascular outcomes in clinical practice (27, 28).

Interestingly, we noted that albuminuria within the normal range remained associated with T2DM in our study. Participants with abnormal albuminuria (UACR ≥30 mg/g) had a 1.78-fold risk of T2DM than those with the normal range (UACR <30 mg/g) (*p* < 0.0001). When UACR was within the normal range, participants with high normal albuminuria (the fifth quintile) were still more likely to have the incidence of T2DM (OR = 2.00 (95% CI = 1.65-2.44), *p* < 0.0001), and the association between high normal albuminuria and T2DM was more significant in participants without HTN and CVDs. This difference may be explained, in part, by the smaller sample size of the HTN and CVD group than the non-HTN and CVD-free group in our study. Further large sample and prospective studies are necessary to clarify the association between UACR and the incidence of T2DM in different subgroups of blood pressure and CVDs.

More importantly, it is well known that albuminuria is an established risk factor for CVD morbidity and mortality both in diabetic and hypertensive individuals. In the national and international guidelines, albuminuria is recommended as a routine screening parameter in individuals at high risk for CVDs (29–31). Also, it has been recognized as a significant indicator of the incidence of generalized atherosclerosis because of the close association of albuminuria with atherosclerotic risk factors and microvascular endothelial damage (32). Findings from population-based studies have reported a significant relationship between albuminuria and CVDs (27, 28). Studies on individuals without T2DM and HTN also reached similar conclusions, which was in line with our findings (33). A prospective study, including 2,484 white subjects, found that nondiabetic individuals with albuminuria have a 1.38-fold increased risk of cardiovascular mortality after adjustment for a wide spectrum of risk factors, and a markedly high 5.68-fold increased risk of cardiovascular mortality was observed in the diabetic population (34). This significant association was also assessed in the general population in this research. Additionally, a study of 40,548 individuals found that a 2-fold increase in albuminuria conferred a 1.29-fold increased risk of cardiovascular mortality (35). The results of our study, which showed an association between abnormal UACR and CVDs in the general population in seven regions across China, agree with the earlier ones. Participants with abnormal albuminuria (UACR ≥30 mg/g) had a 1.12-fold increased risk of CVDs than those with normal albuminuria in our study.

Our study noted that UACR, even within the normal range, exhibited a significant association with CVDs after adjusting for confounding factors. Low-grade albuminuria can predict the incidence of CVDs (6). The Framingham Study, including middle-aged nonhypertensive and nondiabetic individuals with normal UACR, found that low-grade UACR below the abnormal threshold can effectively indicate the development of CVDs (36). Any degree of albuminuria has been proven to be a risk factor for CVDs in diabetic and nondiabetic patients. The risk increases with albuminuria, even below the microalbuminuria cutoff (28). Every 3.5 mg/g increment in UACR conferred a 5.9% increased risk of CVDs after adjustment for age and sex (28). Arnlov et al. proposed a nearly 3-fold increased risk of CVDs in people without HTN and T2DM but with UACR ≥3.9 mg/g in men and ≥7.5 mg/g in women, which was equal to the sex-specific median value (36).

CVD events have been pronounced to be predictable by UACR variation within the normal range (37). The discrepancies between UACR within the normal range and CVDs in different subgroups might be accountable for the interaction of stratification variables with CVDs. It is documented that UACR was significantly associated with components of metabolic syndrome, including blood glucose, pressure, and lipid levels (38). ACC/AHA and ESC/EAS guidelines have recommended LDL-C to be the most crucial risk lipid factor and therapeutic goal for CVDs (39), and the association between UACR within the normal range and CVDs in participants with dyslipidemia was at the borderline significant level in our study. It is well noticed that despite the achieving of optimal LDL-C level, an alarming number of CVD events still occur in clinical practice (40, 41). The contribution of other lipid components and subtractions to CVD development is increasingly being recognized (42, 43). Traditionally, high HDL-C was confirmed to be protective against the incidence and development of atherosclerosis, and low HDL-C was associated with an increased risk of CVDs (44). However, recent clinical trials reported that low HDL-C is not a cause of atherosclerosis, as originally thought, but renewed interest in elevated TG has been generated (45). Some studies supported the theory that elevated TG has a remarkable association with increased risk of CVDs (46, 47).

Moreover, reports from the CACTI Study pointed out that TG independently predicted increased odds of both related CVDs and albuminuria in patients with diabetes. Apart from this, several studies placed great importance on the average levels and ideal targets of glycemic parameters, and it was shown that individuals with CVDs can benefit from well control of blood glucose (48, 49). Although elevated glucose parameters have been treated as modifiable cardiovascular risk factors and robust predictors of CVDs, HbA1c serves as a superior indicator of cardiovascular events than FBG and PBG in clinical practice (50); this might be accountable for the fluctuation of FBG and PBG in different individuals, which could be influenced by various factors. Lots of research has been carried out on the relationship between HTN and CVDs. The relationship between blood pressure and the increased risk of CVDs has been reported to be graded and continuous, starting from 115/75 mmHg, well within what is thought to be the normotensive range (51). It is of great importance to comprehensively consider the predicted risk of atherosclerotic CVDs rather than the level of blood pressure alone, as patients with high CVD risk could derive the benefits from blood pressure-lowering treatment (52). Moreover, an association has also been reported between albuminuria, stroke, and peripheral vascular diseases in several studies (6, 53, 54). Albuminuria may occur due to vascular damage, indicating systemic endothelial dysfunction. The abovementioned evidence may further support our findings. Thus, early identification and prevention of albuminuria are of great significance and could reduce the risk of CVDs.

## Limitations

Our study was a multicenter study based on a seven-region community population, which representatively demonstrates the distribution of different regions across China. However, there are still limitations in our study. First, the variables in our study were measured at the same time. Since this is an observational study and cross-sectional analysis, there may be limitations in concluding causal relationships. It is really true that the causal relationship between albuminuria and the abovementioned diseases cannot be determined in our study. Thus, the association of UACR with HTN, T2DM, HTN with T2DM, dyslipidemia, and CVDs should be further explored in follow-up studies. It is vital to investigate the long-term effects of high normal levels of albuminuria on the risk of cardiovascular outcome and mortality. Second, because the elderly population was from China, the association among other ethnic populations needed to be confirmed, and the results may not apply to young people. Third, although the participants using ACEI/ARB were excluded in our study, the possibility that other medications may partially influence the association could not be eliminated. However, the association of UACR with HTN, T2DM, HTN with T2DM, dyslipidemia, and CVDs persisted after adjusting for antihypertensive drugs, hypoglycemic drugs, and lipid-lowering drugs in multivariable regression analysis. Finally, the urinary albumin excretion was evaluated based on a spot morning. It is undeniable that multiple samples could provide more stable results of albuminuria. However, it has been reported that the results of spot urine samples have a close association with those of 24h and multiple urine samples. Because it is more convenient, the use of spot samples for evaluating UACR has been recommended as a reliable alternative in large epidemiological specimen collection (55). Herein, we emphasize the association between UACR, even within the normal range, and increased risk of HTN, T2DM, HTN with T2DM, dyslipidemia, and CVDs, and such people should be vigilant about the detection, avoidance, and intervention of the presence of albuminuria.

## Conclusion

In summary, we observe a higher prevalence of HTN, T2DM, HTN with T2DM, dyslipidemia, and CVDs in abnormal UACR and reveal a significant association of UACR, even within the normal range, with HTN, T2DM, HTN with T2DM, dyslipidemia, and CVDs. Thus, we propose that albuminuria might be a simple and efficient indicator of metabolic diseases as well as CVDs, and targeting the early prevention as well as the intervention of albuminuria metabolism may increase the possibility of successful drug discovery in the field of CVDs and its related diseases.

## Data Availability Statement

The datasets presented in this article are not readily available for the protection of patient privacy. Requests to access the datasets should be directed to the corresponding author muyiming@301hospital.com.cn.

## Ethics Statement 

The studies involving human participants were reviewed and approved by the Committee on Human Research at Rui-Jin Hospital Affiliated with the School of Medicine, Shanghai Jiao Tong University (No. 2014-52). The patients/participants provided their written informed consent to participate in this study.

## Author Contributions

JW, YW, and YL have contributed equally to this work and share first authorship. YM, WG, and ZHL have contributed equally to this work and share corresponding authorship. YM and JW contributed to the conception and design of the study. YL, YW, WW, ZG, LY, GQ, XT, QW, LC, ZJL, WG, ZHL, YH, and LJ recruited the subjects and supervised the study. JW analyzed the data and wrote the initial draft of the paper. YM, JW, YW, YL, WG, and ZHL contributed to the writing, reviewing, and revising of the manuscript. All authors have read and approved the final manuscript.

## Conflict of Interest

The authors declare that the research was conducted in the absence of any commercial or financial relationships that could be construed as a potential conflict of interest.

## Publisher’s Note

All claims expressed in this article are solely those of the authors and do not necessarily represent those of their affiliated organizations, or those of the publisher, the editors and the reviewers. Any product that may be evaluated in this article, or claim that may be made by its manufacturer, is not guaranteed or endorsed by the publisher.

## Abbreviations

ALT, alanine aminotransferase; AST, aspartate aminotransferase; BMI, body mass index (BMI); CHD, coronary heart disease; Cr, creatinine; CKD-EPI, Chronic Kidney Disease Epidemiology Collaboration; 95% CI, 95% confidence intervals; CVDs, cardiovascular diseases; DBP, diastolic blood pressure; DKD, diabetic kidney disease; FPG, fasting plasma glucose; eGFR, estimated glomerular filtration rate; HbA1c, hemoglobin A1c; HDL-C, high-density lipoprotein cholesterol; HR, heart rate; HTN, hypertension; LDL-C, low-density lipoprotein cholesterol; MI, myocardial infarction; OR, odds ratio; PBG, 2 h postload blood glucose; SBP, systolic blood pressure; SD, standard deviation; TC, total cholesterol; TG, triglyceride; T2DM, type 2 diabetes mellitus; UACR, urine albumin to creatinine ratio; WC, waist circumference.
