# Supplementary material for: High Normal Urinary Albumin–Creatinine Ratio Is Associated With Hypertension, Type 2 Diabetes Mellitus, HTN With T2DM, Dyslipidemia, and Cardiovascular Diseases in the Chinese Population: A Report From the REACTION Study
Source: Front Endocrinol (Lausanne). 2022 May 20;13:864562. doi: 10.3389/fendo.2022.864562 (PMC9165688; doi:10.3389/fendo.2022.864562)
Supplement: Supplementary file 1 [file Table_1.docx]

**Table S1 Characteristics of study population by HTN category**

| HTN | No | Yes | P-value |
| --- | --- | --- | --- |
| N | 22802 | 17386 |  |
| Age | 55.50 (50.32-60.69) | 60.85 (55.37-68.08) | <0.001 |
| BMI | 23.53 (21.50-25.72) | 25.39 (23.28-27.69) | <0.001 |
| ALT | 14.00 (10.00-20.00) | 16.00 (12.00-22.00) | <0.001 |
| AST | 20.00 (17.00-24.00) | 21.00 (18.00-26.00) | <0.001 |
| SBP | 120.00(110.00-128.00) | 147.00(140.00-159.00) | <0.001 |
| DBP | 73.00 (67.00-78.00) | 84.00 (76.00-91.00) | <0.001 |
| HR | 77.00 (71.00-85.00) | 78.00 (71.00-87.00) | <0.001 |
| TC | 4.97 (4.27-5.71) | 5.16 (4.43-5.90) | <0.001 |
| TG | 1.27 (0.92-1.82) | 1.53 (1.09-2.18) | <0.001 |
| LDL-C | 2.88 (2.32-3.48) | 3.01 (2.43-3.63) | <0.001 |
| HDL-C | 1.32 (1.11-1.55) | 1.26 (1.07-1.48) | <0.001 |
| FBG | 5.40 (5.01-5.90) | 5.77 (5.29-6.58) | <0.001 |
| PBG | 6.95 (5.76-8.79) | 8.21 (6.53-11.20) | <0.001 |
| HbA1c | 5.80 (5.50-6.20) | 6.00 (5.70-6.40) | <0.001 |
| eGFR | 96.80(93.11-100.44) | 93.01 (88.35-96.85) | <0.001 |
| UACR | 8.99 (5.48-16.74) | 12.31 (6.72-25.26) | <0.001 |
| Sex |  |  | <0.001 |
| men | 6290 (27.59%) | 5933 (34.13%) |  |
| women | 16512 (72.41%) | 11453 (65.87%) |  |
| Smoking |  |  | <0.001 |
| No | 19311 (84.69%) | 14976 (86.14%) |  |
| Occasional | 747 (3.28%) | 462 (2.66%) |  |
| Frequently | 2744 (12.03%) | 1948 (11.20%) |  |
| Drinking |  |  | <0.001 |
| No | 16822 (73.77%) | 13314 (76.58%) |  |
| Occasional | 4723 (20.71%) | 2734 (15.73%) |  |
| Frequently | 1257 (5.51%) | 1338 (7.70%) |  |
| Antihypertensive drugs |  |  | <0.001 |
| Yes | 206 (0.90%) | 6246 (35.93%) |  |
| No | 22596 (99.10%) | 11140 (64.07%) |  |
| Hypoglycemic drugs |  |  | <0.001 |
| Yes | 1329 (5.83%) | 2447 (14.07%) |  |
| No | 21473 (94.17%) | 14939 (85.93%) |  |
| T2DM |  |  | <0.001 |
| No | 20476 (89.80%) | 13507 (77.69%) |  |
| Yes | 2326 (10.20%) | 3879 (22.31%) |  |
| HTN |  |  | <0.001 |
| No | 22802 (100.00%) | 0 (0.00%) |  |
| Yes | 0 (0.00%) | 17386 (100.00%) |  |
| CVDs |  |  | <0.001 |
| No | 22181 (97.28%) | 15782 (90.77%) |  |
| Yes | 621 (2.72%) | 1604 (9.23%) |  |
| Dyslipidemia |  |  | <0.001 |
| No | 14236 (62.43%) | 8935 (51.39%) |  |
| Yes | 8566 (37.57%) | 8451 (48.61%) |  |
| HTN with T2DM |  |  | <0.01 |
| No | 22802 (100.00%) | 13507 (77.69%) |  |
| Yes | 0 (0.00%) | 3879 (22.31%) |  |

Data were mean ± SD or median (Q1-Q3) for non-normal distribution of variables or numbers (%) for categorical variables

BMI: body mass index; SBP: systolic blood pressure; DBP: diastolic blood pressure; ALT: alanine transferase; AST: aspartate transferase; HR: hearts rate; TG: triglyceride; TC: high cholesterol; LDL-C: low-density lipoprotein cholesterol; HDL-C: high-density lipoprotein cholesterol; FBG: fasting plasma glucose; PBG: 2 h post-load blood glucose; HbA1c: glycosylated hemoglobin; eGFR: estimated glomerular filtration rate; T2DM:type 2 diabetes mellitus; CVDs: cardiovascular diseases; UACR: urinary albumin to creatinine ratio

**
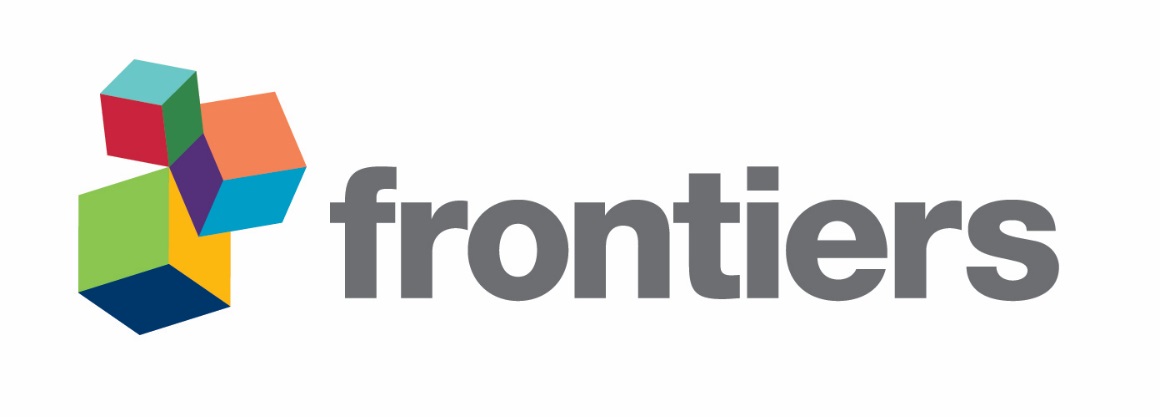
**
