# Supplementary material for: High Normal Urinary Albumin–Creatinine Ratio Is Associated With Hypertension, Type 2 Diabetes Mellitus, HTN With T2DM, Dyslipidemia, and Cardiovascular Diseases in the Chinese Population: A Report From the REACTION Study
Source: Front Endocrinol (Lausanne). 2022 May 20;13:864562. doi: 10.3389/fendo.2022.864562 (PMC9165688; doi:10.3389/fendo.2022.864562)
Supplement: Supplementary file 2 [file Table_2.docx]

**Table S2 Characteristics of study population by T2DM category**

| T2DM | No | Yes | P-value |
| --- | --- | --- | --- |
| N | 33983 | 6205 |  |
| Age | 56.99 (51.88-62.97) | 61.09 (55.46-68.50) | <0.001 |
| BMI | 24.14 (22.00-26.43) | 25.30 (23.22-27.72) | <0.001 |
| ALT | 15.00 (11.00-20.00) | 17.00 (12.00-24.00) | <0.001 |
| AST | 20.00 (17.00-25.00) | 20.00 (16.00-25.00) | <0.001 |
| SBP | 128.00(116.00-143.00) | 138.00 (125.00-153.00) | <0.001 |
| DBP | 76.00 (70.00-84.00) | 78.00 (71.00-85.00) | <0.001 |
| HR | 77.00 (70.00-85.00) | 80.00 (72.00-88.00) | <0.001 |
| TC | 5.06 (4.34-5.78) | 5.06 (4.29-5.84) | 0.505 |
| TG | 1.33 (0.96-1. 91) | 1.66 (1.15-2.41) | <0.001 |
| LDL-C | 2.94 (2.37-3.55) | 2.92 (2.33-3.55) | 0.045 |
| HDL-C | 1.31 (1.11-1.54) | 1.20 (1.02-1.40) | <0.001 |
| FBG | 5.41 (5.05-5.85) | 7.90 (7.00-9.58) | <0.001 |
| PBG | 7.00 (5.82-8.54) | 14.28 (11.63-17.73) | <0.001 |
| HbA1c | 5.80 (5.50-6.10) | 7.20 (6.50-8.30) | <0.001 |
| eGFR | 95.73 (91.50-99.42) | 92.77 (88.14-96.68) | <0.001 |
| UACR | 9.60 (5.72-18.42) | 15.20 (7.68-31.26) | <0.001 |
| Sex |  |  | <0.001 |
| men | 9800 (28.84%) | 2423 (39.05%) |  |
| women | 24183 (71.16%) | 3782 (60.95%) |  |
| Smoking |  |  | <0.001 |
| No | 29089 (85.60%) | 5198 (83.77%) |  |
| Occasional | 1002 (2.95%) | 207 (3.34%) |  |
| Frequently | 3892 (11.45%) | 800 (12.89%) |  |
| Drinking |  |  | <0.001 |
| No | 25351 (74.60%) | 4785 (77.12%) |  |
| Occasional | 6467 (19.03%) | 990 (15.95%) |  |
| Frequently | 2165 (6.37%) | 430 (6.93%) |  |
| Antihypertensive drugs |  |  | <0.001 |
| Yes | 4793 (14.10%) | 1659 (26.74%) |  |
| No | 29190 (85.90%) | 4546 (73.26%) |  |
| Hypoglycemic drugs |  |  | <0.001 |
| Yes | 32 (0.09%) | 3744 (60.34%) |  |
| No | 33951 (99.91%) | 2461 (39.66%) |  |
| T2DM |  |  | <0.001 |
| No | 33983 (100.00%) | 0 (0.00%) |  |
| Yes | 0 (0.00%) | 6205 (100.00%) |  |
| HTN |  |  | <0.001 |
| No | 20476 (60.25%) | 2326 (37.49%) |  |
| Yes | 13507 (39.75%) | 3879 (62.51%) |  |
| CVDs |  |  | <0.001 |
| No | 32418 (95.39%) | 5545 (89.36%) |  |
| Yes | 1565 (4.61%) | 660 (10.64%) |  |
| Dyslipidemia |  |  | <0.001 |
| No | 20350 (59.88%) | 2821 (45.46%) |  |
| Yes | 13633 (40.12%) | 3384 (54.54%) |  |
| HTN with T2DM |  |  | <0.01 |
| No | 33983 (100.00%) | 2326 (37.49%) |  |
| Yes | 0 (0.00%) | 3879 (62.51%) |  |

Data were mean ± SD or median (Q1-Q3) for non-normal distribution of variables or numbers (%) for categorical variables

BMI: body mass index; SBP: systolic blood pressure; DBP: diastolic blood pressure; ALT: alanine transferase; AST: aspartate transferase; HR: hearts rate; TG: triglyceride; TC: high cholesterol; LDL-C: low-density lipoprotein cholesterol; HDL-C: high-density lipoprotein cholesterol; FBG: fasting plasma glucose; PBG: 2 h post-load blood glucose; HbA1c: glycosylated hemoglobin; eGFR: estimated glomerular filtration rate; T2DM:type 2 diabetes mellitus; CVDs: cardiovascular diseases; UACR: urinary albumin to creatinine ratio

**
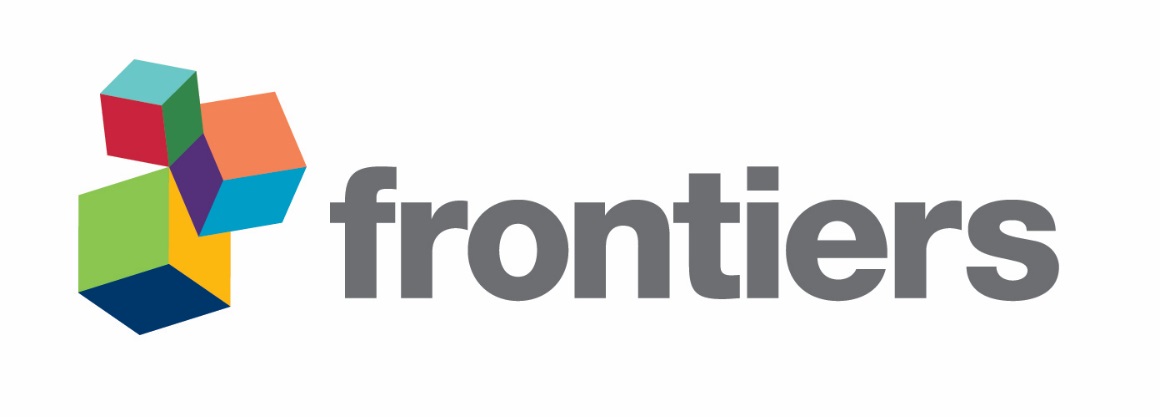
**
