# Supplementary material for: High Normal Urinary Albumin–Creatinine Ratio Is Associated With Hypertension, Type 2 Diabetes Mellitus, HTN With T2DM, Dyslipidemia, and Cardiovascular Diseases in the Chinese Population: A Report From the REACTION Study
Source: Front Endocrinol (Lausanne). 2022 May 20;13:864562. doi: 10.3389/fendo.2022.864562 (PMC9165688; doi:10.3389/fendo.2022.864562)
Supplement: Supplementary file 3 [file Table_3.docx]

**Table S3 Characteristics of study population by HTN with T2DM category**

| HTN with T2DM | No | Yes | P-value |
| --- | --- | --- | --- |
| N | 36309 | 3879 |  |
| Age | 57.07 (51.93-63.09) | 62.95 (57.13-70.18) | <0.001 |
| BMI | 24.16 (22.03-26.45) | 25.81 (23.82-28.18) | <0.001 |
| ALT | 15.00 (11.00-21.00) | 17.00 (12.00-25.00) | <0.001 |
| AST | 20.00 (17.00-25.00) | 20.00 (17.00-26.00) | <0.001 |
| SBP | 128.00 (116.00-141.00) | 149.00 (140.00-162.00) | <0.001 |
| DBP | 76.00 (70.00-83.00) | 81.00 (74.00-90.00) | <0.001 |
| HR | 78.00 (71.00-85.00) | 80.00 (72.00-89.00) | <0.001 |
| TC | 5.05 (4.33-5.78) | 5.12 (4.34-5.92) | <0.001 |
| TG | 1.34 (0.96-1.93) | 1.71 (1.21-2.46) | <0.001 |
| LDL-C | 2.93 (2.37-3.55) | 2.96 (2.35-3.61) | 0.080 |
| HDL-C | 1.30 (1.10-1.53) | 1.20 (1.02-1.40) | <0.001 |
| FBG | 5.47 (5.09-5.96) | 7.87 (7.00-9.40) | <0.001 |
| PBG | 7.14 (5.90-8.95) | 14.26 (11.71-17.64) | <0.001 |
| HbA1c | 5.80 (5.60-6.20) | 7.20 (6.50-8.20) | <0.001 |
| eGFR | 95.67 (91.43-99.33) | 91.52 (87.17-95.44) | <0.001 |
| UACR | 9.74 (5.76-18.81) | 17.39 (8.46-37.10) | <0.001 |
| Sex |  |  | <0.001 |
| men | 10770 (29.66%) | 1453 (37.46%) |  |
| women | 25539 (70.34%) | 2426 (62.54%) |  |
| Smoking |  |  | 0.110 |
| No | 30934 (85.20%) | 3353 (86.44%) |  |
| Occasional | 1104 (3.04%) | 105 (2.71%) |  |
| Frequently | 4271 (11.76%) | 421 (10.85%) |  |
| Drinking |  |  | <0.001 |
| No | 27087 (74.60%) | 3049 (78.60%) |  |
| Occasional | 6906 (19.02%) | 551 (14.20%) |  |
| Frequently | 2316 (6.38%) | 279 (7.19%) |  |
| Antihypertensive drugs |  |  | <0.001 |
| Yes | 4837 (13.32%) | 1615 (41.63%) |  |
| No | 31472 (86.68%) | 2264 (58.37%) |  |
| Hypoglycemic drugs |  |  | <0.001 |
| Yes | 1346 (3.71%) | 2430 (62.65%) |  |
| No | 34963 (96.29%) | 1449 (37.35%) |  |
| T2DM |  |  | <0.001 |
| No | 33983 (93.59%) | 0 (0.00%) |  |
| Yes | 2326 (6.41%) | 3879 (100.00%) |  |
| HTN |  |  | <0.001 |
| No | 22802 (62.80%) | 0 (0.00%) |  |
| Yes | 13507 (37.20%) | 3879 (100.00%) |  |
| CVDs |  |  | <0.001 |
| No | 34621 (95.35%) | 3342 (86.16%) |  |
| Yes | 1688 (4.65%) | 537 (13.84%) |  |
| Dyslipidemia |  |  | <0.001 |
| No | 21497 (59.21%) | 1674 (43.16%) |  |
| Yes | 14812 (40.79%) | 2205 (56.84%) |  |

Data were mean ± SD or median (Q1-Q3) for non-normal distribution of variables or numbers (%) for categorical variables

BMI: body mass index; SBP: systolic blood pressure; DBP: diastolic blood pressure; ALT: alanine transferase; AST: aspartate transferase; HR: hearts rate; TG: triglyceride; TC: high cholesterol; LDL-C: low-density lipoprotein cholesterol; HDL-C: high-density lipoprotein cholesterol; FBG: fasting plasma glucose; PBG: 2 h post-load blood glucose; HbA1c: glycosylated hemoglobin; eGFR: estimated glomerular filtration rate; T2DM:type 2 diabetes mellitus; CVDs: cardiovascular diseases; UACR: urinary albumin to creatinine ratio

**
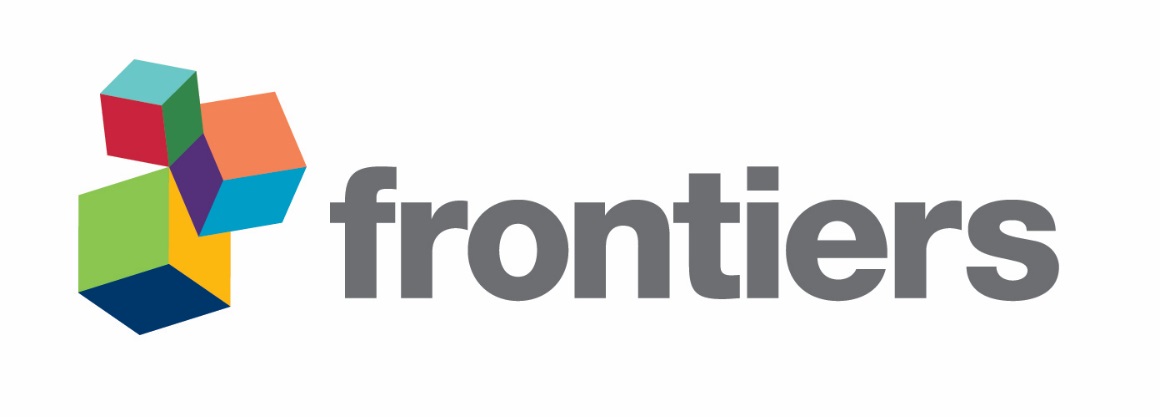
**
