# Supplementary material for: High Normal Urinary Albumin–Creatinine Ratio Is Associated With Hypertension, Type 2 Diabetes Mellitus, HTN With T2DM, Dyslipidemia, and Cardiovascular Diseases in the Chinese Population: A Report From the REACTION Study
Source: Front Endocrinol (Lausanne). 2022 May 20;13:864562. doi: 10.3389/fendo.2022.864562 (PMC9165688; doi:10.3389/fendo.2022.864562)
Supplement: Supplementary file 4 [file Table_4.docx]

**Table S4 Characteristics of study population by dyslipidemia category**

| Dyslipidemia | No | Yes | P-value |
| --- | --- | --- | --- |
| N | 23171 | 17017 |  |
| Age | 56.99 (51.74-63.55) | 58.28 (53.36-64.44) | <0.001 |
| BMI | 23.81 (21.67-26.13) | 24.98 (22.94-27.24) | <0.001 |
| ALT | 14.00 (11.00-20.00) | 16.00 (11.00-23.00) | <0.001 |
| AST | 20.00 (17.00-25.00) | 21.00 (17.00-25.00) | <0.001 |
| SBP | 127.00(115.00-142.00) | 132.00 (120.00-147.00) | <0.001 |
| DBP | 76.00 (69.00-83.00) | 78.00 (71.00-85.00) | <0.001 |
| HR | 77.00 (70.00-85.00) | 78.00 (71.00-86.00) | <0.001 |
| TC | 4.93 (4.37-5.45) | 5.47 (4.23-6.45) | <0.001 |
| TG | 1.16 (0.87-1.52) | 2.00 (1.30-2.79) | <0.001 |
| LDL-C | 2.89 (2.41-3.34) | 3.08 (2.28-4.07) | <0.001 |
| HDL-C | 1.37 (1.21-1.57) | 1.11 (0.94-1.40) | <0.001 |
| FBG | 5.44 (5.05-6.00) | 5.70 (5.23-6.49) | <0.001 |
| PBG | 7.03 (5.80-9.03) | 8.04 (6.42-10.82) | <0.001 |
| HbA1c | 5.80 (5.50-6.20) | 6.00 (5.70-6.40) | <0.001 |
| eGFR | 95.73 (91.17-99.62) | 94.75 (90.62-98.29) | <0.001 |
| UACR | 9.75 (5.75-19.05) | 10.80 (6.16-21.52) | 0.114 |
| Sex |  |  | <0.001 |
| men | 6365 (27.47%) | 5858 (34.42%) |  |
| women | 16806 (72.53%) | 11159 (65.58%) |  |
| Smoking |  |  | <0.001 |
| No | 20220 (87.26%) | 14067 (82.66%) |  |
| Occasional | 615 (2.65%) | 594 (3.49%) |  |
| Frequently | 2336 (10.08%) | 2356 (13.84%) |  |
| Drinking |  |  | 0.008 |
| No | 17480 (75.44%) | 12656 (74.37%) |  |
| Occasional | 4263 (18.40%) | 3194 (18.77%) |  |
| Frequently | 1428 (6.16%) | 1167 (6.86%) |  |
| Antihypertensive drugs |  |  | <0.001 |
| Yes | 3144 (13.57%) | 3308 (19.44%) |  |
| No | 20027 (86.43%) | 13709 (80.56%) |  |
| Hypoglycemic drugs |  |  | <0.001 |
| Yes | 1806 (7.79%) | 1970 (11.58%) |  |
| No | 21365 (92.21%) | 15047 (88.42%) |  |
| T2DM |  |  | <0.001 |
| No | 20350 (87.83%) | 13633 (80.11%) |  |
| Yes | 2821 (12.17%) | 3384 (19.89%) |  |
| HTN |  |  | <0.001 |
| No | 14236 (61.44%) | 8566 (50.34%) |  |
| Yes | 8935 (38.56%) | 8451 (49.66%) |  |
| CVDs |  |  | <0.001 |
| No | 22028 (95.07%) | 15935 (93.64%) |  |
| Yes | 1143 (4.93%) | 1082 (6.36%) |  |
| HTN with T2DM |  |  | <0.01 |
| No | 21497 (92.78%) | 14812 (87.04%) |  |
| Yes | 1674 (7.22%) | 2205 (12.96%) |  |

Data were mean ± SD or median (Q1-Q3) for non-normal distribution of variables or numbers (%) for categorical variables

BMI: body mass index; SBP: systolic blood pressure; DBP: diastolic blood pressure; ALT: alanine transferase; AST: aspartate transferase; HR: hearts rate; TG: triglyceride; TC: high cholesterol; LDL-C: low-density lipoprotein cholesterol; HDL-C: high-density lipoprotein cholesterol; FBG: fasting plasma glucose; PBG: 2 h post-load blood glucose; HbA1c: glycosylated hemoglobin; eGFR: estimated glomerular filtration rate; T2DM:type 2 diabetes mellitus; CVDs: cardiovascular diseases; UACR: urinary albumin to creatinine ratio

**
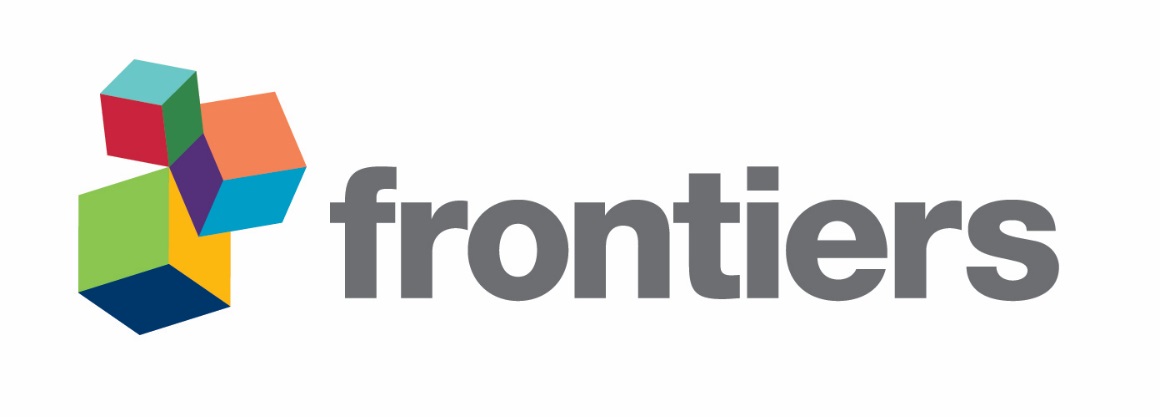
**
